# Species Identification, Virulence Factors, and Antifungal Resistance in Clinical *Candida* Isolates from ICU Patients

**DOI:** 10.3390/microorganisms14010241

**Published:** 2026-01-21

**Authors:** Paola Aparecida Alves Ferreira, Lucas Daniel Cibolli Roso, Daniel Almeida Freitas, Ana Paula Pereira Bressani, Paulo Henrique da Cruz Ferreira, Emerson Cotta Bodevan, Cristiane Rocha Fagundes Moura, Rosane Freitas Schwan, Vanessa Amaral Mendonça, Karina Teixeira Magalhães-Guedes, Cíntia Lacerda Ramos

**Affiliations:** 1 Department of Basic Sciences, Federal University of Vale of Jequitinhonha and Mucuri (UFVJM), Street MGT 367—Km 583, no 5000, Alto da Jacuba, Diamantina 39100-000, MG, Brazil; 2 Post-Graduate Program in Agricultural Microbiology, Department of Biology, Federal University of Lavras, Lavras 37200-900, MG, Brazil; 3 Post-Graduate Program in Chemistry Engineering, Federal University of Bahia (UFBA), Street Professor Aristídes Novis, 02, Federação, Salvador 40210-630, BA, Brazil; 4 Post-Graduate Program in Food Science, Federal University of Bahia (UFBA), Barão of Geremoabo Street, s/n, Ondina, Salvador 40171-970, BA, Brazil

**Keywords:** *Candida albicans*, *Candida* non-*albicans*, identification, MALDI-TOF, antifungal resistance, virulence factors

## Abstract

*Candida* spp. are important opportunistic human fungal pathogens. This study aimed to identify and characterize *Candida* spp. obtained from patients admitted to an Intensive Care Unit (ICU), focusing on virulence attributes and susceptibility to antifungal agents. A total of 131 isolates from oral and tracheobronchial secretions of adult ICU patients were evaluated. Phenotypic identification was performed using chromogenic culture media for *Candida*, followed by MALDI-TOF mass spectrometry, with representative isolates confirmed by ITS sequencing. Antifungal susceptibility to fluconazole, ketoconazole, and amphotericin B was determined only by the agar disk diffusion method, and virulence was assessed through esterase, DNase, protease, and hemolytic activity assays. *C. albicans* was the prevalent species, followed by *C. tropicalis*, *C. krusei*, *C. glabrata*, *C. parapsilosis*, *C. dubliniensis*, *C. lusitaniae*, and *C. guilliermondii*. Antifungal resistance rates reached 51.1% for fluconazole, 42.7% for ketoconazole, and 19.1% for amphotericin B, as determined by disk diffusion method. Overall, 64.9% of the isolates exhibited esterase activity, 18.3% DNase, 45.8% protease, and 67.2% exhibited hemolytic activity. Oral isolates were more frequent than tracheal isolates and demonstrated a higher prevalence of antifungal resistance and virulence traits. These findings underscore the epidemiological importance of characterizing *Candida* species in hospitals to better understand the yeast profile and to support adequate clinical management.

## 1. Introduction

The genus *Candida* is considered one of the most common opportunistic human fungal pathogens, possessing several species with enormous phylogenetic and phenotypic diversity. It represents the leading cause of mucosal fungal infections. In critically ill or severely immunocompromised patients, invasive candidiasis is associated with high mortality rates, particularly in intensive care settings [1].

Several factors play important roles in the pathogenicity and virulence of *Candida* spp. A virulence factor is the attribute that determines the ability of strains to cause disease through specific molecules, mainly proteins and enzymes, produced and released with the aim of circumventing the host’s immune system defense mechanisms [2]. *Candida*’s extracellular hydrolytic/lipolytic enzymes (proteinases, phospholipases, DNases, hemolysins, and lipases) facilitate tissue penetration, and adhesins and invasins are responsible for mediating adhesion and invasion, while heat shock proteins “HSPs” and cytolytic proteins are equally harmful to the host cell [3].

Understanding the antifungal resistance profile of *Candida* spp. helps in selecting the most appropriate antifungal medication, thereby reducing the rates of antifungal resistance [2]. *Candida* spp. infections are typically treated with existing conventional antifungals, including azoles and polyenes. However, in recent decades, as with antibiotics, the incorrect use of antifungal drugs has led to the development of drug-resistant clinical isolates, rendering clinically available drugs ineffective [4]. Therefore, there is a significant demand for studies to develop new therapies to combat pathogenic fungi, as well as to predict and prevent the evolution of resistance to the current commercial antifungals [4].

A crucial point to emphasize is that identifying *Candida* yeasts at the species level is essential for obtaining a timely diagnosis and appropriate treatment for these infections. For this reason, several methods of yeast identification have been employed, based on phenotypic, biochemical, immunological, genetic, and spectroscopic techniques [5]. The use of chromogenic medium has been a basic diagnostic tool for yeast identification. It can differentiate *Candida* species through changes in the color and texture of colonies, either by pH indicators and fermentation of specific compounds or chromogenic substances. By using this medium, some species, such as *C. albicans*, *C. tropicalis*, *C. krusei*, *C. dubliniensis*, and, depending on the formulation, *C. glabrata*, can be easily isolated and identified [6]. However, the medium’s specificity is limited, and other identification methods have been used for a more reliable identification, such as matrix-assisted laser desorption/ionization mass spectrometry (MALDI-TOF) or DNA sequencing [5,6]. These approaches are effective in identifying fungi, and the accuracy of both methods depends on the database used.

This study aimed to identify *Candida* species isolated from hospitalized patients in an adult Intensive Care Unit (ICU), using three different identification methods: chromogenic culture medium for *Candida*, MALDI-TOF, and DNA sequencing. The presence of different virulence factors and antifungal resistance of *Candida* isolates were also evaluated.

## 2. Materials and Methods

### 2.1. Yeasts and Growth Conditions

A total of 131 *Candida* strains, previously isolated from oral and tracheobronchial secretions of patients admitted to an adult ICU in Diamantina, Brazil [7], were used. The strains, stored at −80 °C, were reactivated in YEPG (Biolog, Porto Alegre, RS, Brazil) broth (1% yeast extract, 2% glucose, 2% peptone) at 37 °C for 48 h. Subsequently, the strains were inoculated onto Petri dishes containing 2% Sabouraud Dextrose Agar (Biolog, Porto Alegre, RS, Brazil) and incubated at 37 °C for 48 h for subsequent analysis. To minimize potential phenotypic variation, isolates were thawed only once prior to experimental assays and were not subjected to repeated freeze–thaw cycles.

### 2.2. Phenotypic Identification of Candida spp. Using Chromogenic Culture Medium

For phenotypic identification, 10 µL of previously reactivated yeast was inoculated onto plates containing CHROMagar (Kasvi, Madrid, Espanha) culture medium and incubated at 37 °C for 48 h. For identification, the colors of the grown colonies were compared to those provided by the manufacturer. The experiment was performed in triplicate.

### 2.3. Candida spp. Identification by MALDI-TOF Analysis

The 131 yeast strains previously cultivated on Sabouraud agar (Biolog, Porto Alegre, RS, Brazil) were transferred to tubes containing 300 μL of deionized water, vortexed for 30 s, and then followed by the addition of 900 μL of pure ethanol. The suspension was vortexed again for 30 s and then centrifuged for 2 min at 13,000 rpm. Then, 50 μL of 70% formic acid and 50 μL of acetonitrile were added to the precipitate, which was then vortexed and centrifuged for 2 min at 13,000 rpm. The precipitates obtained from the lysed cells were placed in 96-well MALDI flex plates (Bruker Daltonics, Bremen, Germany) containing a polymeric matrix. Complete evaporation of the liquid was then awaited, after which the plates were inserted into the equipment for analysis.

The *Escherichia coli* K12 strain obtained from the culture collection of the University of Minho, Portugal (MUM, http://www.micoteca.deb.uminho.pt, accessed on 9 August 2025) was used for protein extraction, which was then used as a standard for external calibration of the MALDI-TOF. The analyses were performed on the MALDI-TOF microflex LT spectrometer (Bruker Daltonics, Bremen, Germany), using the automated MALDI Biotyper 3.1 software and library version 3.3.1.2 updated with 5989 entries (Bruker Daltonics, Bremen, Germany). The analyses were performed in triplicate. The next step was to construct the dendrogram using the Biotyperlog (Bruker Daltonics, Bremen, Germany). The software grouped the yeast strains based on the similarity between the isolates, as determined by the spectral patterns obtained, and identified them by comparing the spectra present in the database. The MALDI-TOF system used in this study (Bruker Daltonics, Bremen, Germany) recommends a score ≥2.00 for reliable species identification, states that a score of <2.00–1.70 is reliable for genus identification, and considers scores <1.70 as unreliable.

### 2.4. Candida spp. Identification by ITS Sequencing

Representatives of each species identified by the techniques described above (chromogenic medium (Biolog, Porto Alegre, RS, Brazil) and MALDI-TOF) were selected and subjected to sequencing analysis of the ITS region for confirmation of identification. A total of 17 yeasts were selected, and their DNA was extracted using InstaGene Matrix (Bio-Rad, Hercules, CA, USA) according to the manufacturer’s instructions. Polymerase chain reaction (PCR) was performed using primers ITS1 (50-TCCGTAGGTGAACCTGCGG-30) and ITS4 (50-TCCTCCGCTTATTGATATGC-30). One microliter of DNA extracted from each yeast was added to 30 μL of Taq PCR Master Mix (Qiagen, SP, Brazil), 27 μL of H_2_O, and 1 μL of each primer, ITS 1 and ITS 4. PCR was performed under the following conditions: initial denaturation at 95 °C for 5 min; 30 cycles of denaturation at 95 °C for 30 s, annealing at 52 °C for 30 s, and extension at 72 °C for 1 min; and a final extension at 72 °C for 10 min. The amplified products were confirmed by electrophoresis on 1.2% agarose gel. The PCR products were sent for sequencing at ACTGene Análise Moleculares (Alvorada, RS, Brazil). The obtained sequences were compared with those in the GenBank database using the BLAST algorithm (http://www.ncbi.nlm.nih.gov/BLAST/, accessed on 9 August 2025) to determine species identification [8].

### 2.5. Antifungal Sensitivity of Candida Isolates

The susceptibility of *Candida* strains to the antifungal agents fluconazole, ketoconazole, and amphotericin B (Cecon, São Paulo, SP, Brazil) was evaluated using the agar disk diffusion method. The 131 yeast strains previously grown on Sabouraud agar (Biolog, Porto Alegre, RS, Brazil) were resuspended in saline solution (0.9% NaCl), equivalent to a McFarland 0.5 scale (approximately 1 × 10^6^ CFU/mL), and seeded using a sterile swab onto plates containing Mueller-Hinton agar (Fluka, Darmstadt, Germany). Discs containing the antifungals fluconazole, ketoconazole, and amphotericin B (Cecon, São Paulo, SP, Brazil) were placed on the inoculum, and the plates were incubated at 37 °C for 48 h [9]. The resistance/susceptibility of yeasts to antifungals was evaluated by measuring the inhibition halo formed, according to criteria established by the Clinical and Laboratory Standards Institute [9]. Disk diffusion was used as a screening method; however, interpretive criteria for amphotericin B is limited.

### 2.6. Esterase Production

Yeast cells, previously grown on Sabouraud agar (Biolog, Porto Alegre, RS, Brazil), were suspended in saline solution (0.9% NaCl) until reaching turbidity equivalent to McFarland scale 0.5 (approximately 1 × 10^6^ CFU/mL) and inoculated (10 µL) onto esterase agar medium (1% peptone, 0.5% NaCl, 0.1% CaCl_2_, 0.5% Tween 80, 1.5% agar, pH 6.8). The plates were incubated at 37 °C for 10 days. Results were interpreted as positive if a precipitation zone formed around the colony, or negative in the absence of halo formation [10]. The experiment was performed in duplicate.

### 2.7. DNase Production

For inoculum preparation, cultures of each yeast, previously grown on Sabouraud agar (Biolog, Porto Alegre, RS, Brazil) for 24 h, were suspended in saline solution (0.9% NaCl) to achieve a turbidity equivalent to tube 0.5 on the McFarland scale (approximately 1 × 10^6^ CFU/mL). Then, 10 μL aliquots of each suspension were inoculated at equidistant points in Petri dishes (90 × 15 mm) containing DNase agar (Hexis, São Paulo, SP, Brazil). The plates were incubated at 37 °C for 7 days. After incubation, plates were flooded with HCl solution (1 M), and the appearance of a clear halo around the colony indicates that DNA degradation has occurred. The tests were performed in duplicate, and the results of DNase activity were expressed as positive or negative, depending on the presence or absence of a clear halo around the colony [11].

### 2.8. Protease Production

The protease production of *Candida* strains was determined according to Staib [12] with modifications. Yeast cultures grown on Sabouraud agar (Biolog, Porto Alegre, RS, Brazil) for 24 h and standardized in saline solution (0.9% NaCl) according to tube 0.5 of the McFarland scale (approximately 1 × 10^6^ CFU/mL) were inoculated (10 µL) onto Petri dishes containing protease medium (1% yeast extract, 2% glucose, 2% peptone, 5% casein, and 2% agar). The plates were incubated at 37 °C for 7 days. To reveal the result, the plates were flooded with HCl (1 M) for better visualization of casein degradation. The results were interpreted as positive if a precipitation zone formed around the colony, or negative in the absence of halo formation. The assay was performed in duplicate.

### 2.9. Hemolytic Activity

Hemolytic activity was evaluated according to Rorig et al. [13]. Cultures of each yeast, grown on Sabouraud agar (Biolog, Porto Alegre, RS, Brazil) for 24 h, were resuspended in saline solution (0.9% NaCl), with turbidity equivalent to tube 0.5 of the McFarland scale (approximately 1 × 10^6^ CFU/mL). Aliquots (10 μL) of each suspension were inoculated onto Petri dishes (90 × 15 mm) containing 7% sheep blood agar and incubated at 37 °C for 48 h for hemolytic activity analysis. The results were expressed as: alpha hemolysis (partial or incomplete hemolysis), showing a dark halo around the colony; beta hemolysis (total or complete hemolysis), resulting in a clear and translucent zone around the colony; and gamma hemolysis, in the absence of hemolysis or negative hemolysis [13]. The test was performed in duplicate.

### 2.10. Statistical Analysis

The data obtained after the sample analyses were tabulated and presented using absolute (n) and relative (%) frequencies and analyzed using simple descriptive statistics. The results for virulence factors were qualitative (presence/absence), and no semi-quantitative scoring was applied.

The association between *Candida* species (*C. albicans* vs. *C*. non-*albicans*) and the presence of virulence factors (esterase, DNase, protease, and hemolytic activity) as well as antifungal resistance (fluconazole, ketoconazole, and amphotericin B) profiles were evaluated using the chi-square (χ^2^) test. When the expected number of isolates was <5, Fisher’s exact test was applied. Effect sizes were estimated by calculating odds ratios (OR) with 95% confidence intervals (95% CI). All tests were two-tailed, and a *p* value < 0.05 was considered statistically significant.

Given the exploratory, hypothesis-generating nature of the study and the limited sample sizes across several species, no formal correction for multiple comparisons was applied. Statistical analyses were performed using EpiInfo software (version 7). The 131 strains were randomly obtained from patients with *Candida* colonization and were treated as distinct isolates and considered independent units of observation.

## 3. Results and Discussion

### 3.1. Yeast Identification

**Figure 1:**
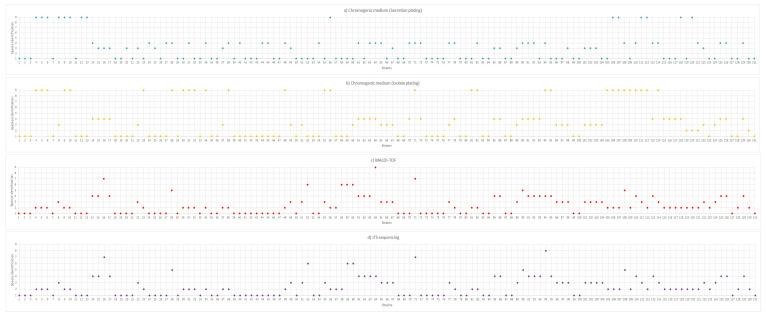
Distribution of *Candida* spp. according to different identification methods: (**a**) from direct secretion plating in chromogenic medium; (**b**) from isolate plating in chromogenic medium; (**c**) by MALDI-TOF analysis; (**d**) by ITS sequencing. Numbers in the *y*-axis correspond to: 1—*C. albicans*; 2—*C. tropicalis*; 3—*C. krusei*; 4—*C. glabrata*; 5—*C. parapsilosis*; 6—*C. dubliniensis*; 7—*C. lusitaniae*; 8—*C. guilliermondii*; 9—No identified.

In this study, 131 *Candida* strains previously isolated from oral (79) and tracheobronchial (52) secretions of patients admitted to an adult ICU in the city of Diamantina, Brazil [7], were identified using three methods: phenotypic analysis using chromogenic culture medium, MALDI-TOF, and sequencing of the ITS region of rDNA. The yeasts were isolated in the previous study [7] involving 135 intubated patients admitted to the ICU from February 2022 to March 2023, of whom 61.5% were male, with a median age of 57.6 years. Most patients (63%) had one or fewer comorbidities. *Candida* species were detected and isolated from tracheal and oral secretions in 35% and 44.4% of the evaluated patients, respectively. It is worth noting that the strains had previously been identified using CHROMagar *Candida* culture medium through direct plating of oral and tracheal secretions, without prior culture and isolation on Sabouraud agar. The distribution of strains according to the species identified by each method is shown in Figure 1.

According to Figure 1, we can observe small discrepancies in the identification of yeasts among the employed methods. The chromogenic method, previously used to identify strains based on direct secretions plating, failed to identify 15 strains due to chromogenic alterations that were incompatible with the culture medium stains. This fact can be attributed to the chromogenic medium used, which is very useful in identifying only five important clinical *Candida* species: *C. tropicalis*, *C. albicans*, *C. krusei*, *C. parapsilosis*, and *C. glabrata*. Another relevant fact is the reaction/interaction of the chromogenic medium with components of oral and tracheal secretions, which can generate positive/negative results. Comparing this identification with the sequencing of the ITS region of rDNA, 72 strains (55.0%) were matched in terms of species identification.

When using the chromogenic medium to identify purified yeasts, it was not possible to identify 29 strains. Initially, the fact that fewer strains were identified compared to those in secretions was surprising. Greater identification efficacy is expected when the yeast is isolated and purified, since there is no interference from components present in the secretions, resulting in clearer colony colors. However, when compared with DNA sequencing, 85 strains (64.9%) were corresponding. Of the 29 unidentified strains, 20 were *C. tropicalis*, which had a grayish color, whereas the colony color should be blue, according to the manufacturer’s manual. Identification using chromogenic culture media is widely employed in clinical studies due to its speed, low cost, and ease of performance [14]. However, studies show that the diagnostic accuracy for the most common isolates is not 100% [15,16], although some studies have reported 100% correspondence [17]. According to Odds [15], some species, such as *C. tropicalis* and *C. krusei*, did not exhibit exclusive coloration after 48 h of incubation, reinforcing the need for other identification methods to achieve more accurate species identification.

**Figure 2:**
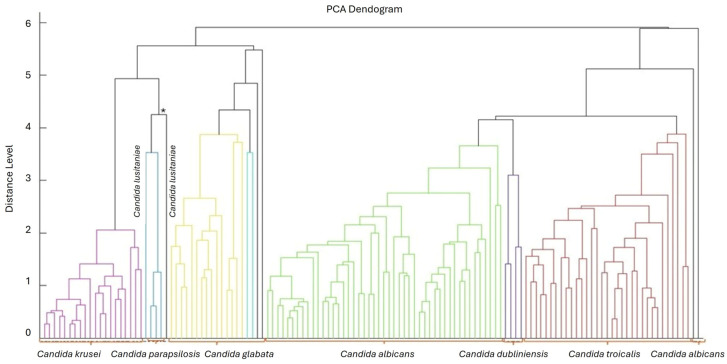
Dendrogram grouping the isolates of *Candida* according to the identified species using the MALDI-TOF technique. * Candida lusitaniae.

In the present study, the MALDI-TOF technique allowed the identification of 110 strains at the species level (84%) (score ≥ 2.00) and 20 strains at the genus level (15.3%) (score < 2.00–1.70), as shown in the dendrogram presented in Figure 2.

The MALDI-TOF technique has been demonstrated to be a promising method for microbial identification, and with the expansion of databases, it can successfully differentiate fungi at the species level [18]. By sequencing the ITS region of rDNA, the identification of 129 strains was confirmed. Correct identification at the species level is extremely important for both effective treatment with the correct therapy, thereby avoiding drug resistance, and for evaluating the emergence of emerging and/or new species [4,6]. Information on endemic species and their pathogenic characteristics in regions of the country is also highly relevant for enhancing knowledge and developing effective treatments, thereby preventing the spread of resistant and virulent strains.

**Figure 3:**
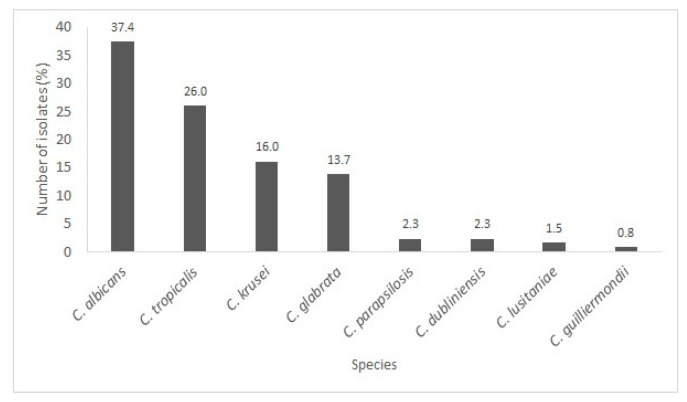
The percentage of isolates of each *Candida* species identified from patients admitted to the ICU.

According to the findings, 131 strains isolated from patients admitted to the ICU were analyzed, and *Candida albicans* predominated (37.4%). The following species were also identified: *Candida tropicalis* (26.0%), *Candida krusei* (16.0%), *Candida glabrata* (13.7%), *Candida parapsilosis* (2.3%), *Candida dubliniensis* (2.3%), *Candida lusitaniae* (1.5%), and *Candida guilliermondii* (0.8%) (Figure 3). Other studies have found *Candida albicans* as the prevalent species in infections [19,20]. A study performed by the SENTRY Antifungal Surveillance Program 2011 analyzed 20,788 *Candida* isolates collected from 135 medical centers in 39 countries between 1997 and 2016 as part of a global surveillance effort. Among these isolates, the majority were *C. albicans* (46.9%). The species *C. glabrata* (18.7%), *C. parapsilosis* (15.9%), *C. tropicalis* (9.3%), *C. krusei* (2.8%), and other species (6.5%), including *C. dubliniensis*, were also identified [1].

The greater ability of *C. albicans* to adhere to mucosa [21] may be related to this yeast being the most frequent cause of candidemia and invasive nosocomial candidiasis [19,22]. However, although *C. albicans* is still the main cause of candidemia, several studies [1,23,24] report a growing number of *C.* non-*albicans* species as causative agents of fungal infections, with a high incidence in adults, associated with high mortality. This condition is primarily due to the emergence of strains resistant to commercial antifungals [1,24].

### Species Present in the Oral and Tracheal Cavities

**Figure 4:**
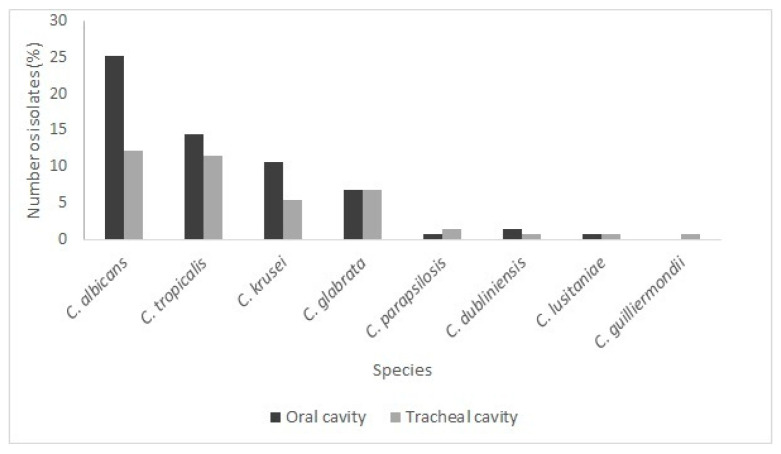
Percentage of isolates of each *Candida* species identified in the oral (black) and tracheal (gray) cavities of patients admitted to the ICU.

From the 131 strains analyzed in this study, 60.3% (79) were isolated from the oral cavities and 39.7% (52) from the tracheal cavities of patients admitted to the ICU and undergoing invasive mechanical ventilation. The percentages of species isolated in each cavity are shown in Figure 4. It was observed that *C. albicans* was the most frequent species in the oral cavity, as demonstrated by other studies, which report that this species corresponds to between 60 and 90% of the species colonizing the oral cavity [25,26,27]. The species *C. tropicalis* represented approximately 14.5%, followed by *C. krusei* (10.7%) and *C. glabrata* (6.9%). Other species appeared less frequently, as observed in other studies [25,26,27].

In general, the oral cavity presented the highest number of isolates of all identified species, except for *C. guilliermondii*, which was identified only in the tracheal cavity, and *C. parapsilosis*, which was more frequent in the tracheal cavity (Figure 4). These two species are commonly found to colonize the skin and are isolated from human hands [28], which may serve as a source of contamination during patient intubation procedures. According to Lima [29], *C. parapsilosis* stands out for its ability to adhere and form biofilms, which favors the colonization of invasive medical devices.

Studies by Govrins & Lass-Flörl [27] demonstrated that among isolates recovered from healthcare professionals who had direct contact with hospitalized patients, *C. parapsilosis* was prevalent. According to these authors, this pathogen is well-adapted to the hospital environment, with the potential for horizontal transmission by healthcare professionals, posing a significant risk of outbreaks in hospital settings. In the present study, the oral and tracheobronchial samples were collected 48 h after intubation. Oral samples were collected before oral hygiene [7]. Clinical data related to *C. guilliermondii* infection are scarce, as this species is an infrequent cause of candidemia and invasive fungal infections. However, it has been associated with decreased susceptibility to triazoles [28]. As this pathogen can colonize human skin, hands, and mucous membranes [30], it has been linked in the literature to diseases such as chronic onychomycosis [31], septic arthritis [32], and endocarditis [33]. Although it is an uncommon pathogen in invasive fungal infections, there have been early reports from 1985 and 2003 that show, since that time, nosocomial transmission through the spread of *C. guilliermondii* in immunocompromised patients [34] and catheter contamination by this pathogen among five surgical patients in an Italian hospital [35].

It is important to note that the detection of *Candida* spp. in oral and tracheal samples does not imply invasive disease and should not be interpreted as evidence of clinical infection.

### 3.2. Antifungal Resistance

The resistance/susceptibility of the 131 yeasts to the commercial antifungals fluconazole, ketoconazole, and amphotericin B was evaluated, and the percentages of isolates resistant to the antifungals are presented in Table 1.

According to Table 1, it is possible to observe that isolates from the oral cavity showed higher resistance values to the three antifungals evaluated compared to those from the tracheal cavity. It is important to highlight that the highest number of yeast isolates was also obtained from this cavity, which may be related to the higher frequency of antifungal resistance. A total of 51.2% of the strains were resistant to fluconazole, with 32.1% isolated from the oral cavity and 19.1% from the tracheal cavity. This high resistance to fluconazole was also reported by other authors [36,37,38]. According to Seneviratne [36], *Candida* spp. is the fourth most frequent microorganism in invasive nosocomial infections and is fluconazole-resistant (including all *Candida* species, mainly *C. albicans*) in most cases. This is a significant concern, as it is the most widely used antifungal agent in the treatment of invasive candidiasis.

Regarding ketoconazole, 42.6% of the isolates showed resistance to this agent. This resistance also poses a significant clinical concern, as it compromises the effectiveness of this widely used azole antifungal, reducing available therapeutic options and may lead to treatment failures, especially in immunocompromised patients. Additionally, there is a risk of cross-resistance with other azoles, such as fluconazole and itraconazole, which further complicates the clinical scenario [33]. The selection of resistant strains can also lead to increased virulence and persistence of infection, thereby increasing hospital morbidity [4].

Amphotericin B showed a lower percentage of resistant strains, 19.2%, being 11.5% from the oral cavity and 7.7% from the tracheal cavity. This result corroborates the data in the literature, which show that *Candida* isolates have reduced levels of resistance to amphotericin B when compared to other antifungals [39]. The resistance rates reported in other studies are very low, approaching zero [4], which may be due to the methods employed for evaluation. The low level of amphotericin B resistance reinforces that, despite its adverse effects and toxicity, this antifungal remains an important agent in the treatment of serious fungal infections. Polyenes, including amphotericin B, are fungicidal, whereas azoles (such as ketoconazole and fluconazole) are fungistatic. This could be the main reason for the increased sensitivity of *Candida* spp. to amphotericin B compared to the other two antifungals [39].

According to Ellepola and colleagues [39], the difference between the polyene (amphotericin B) and the azoles (ketoconazole and fluconazole) may be due to the difference in the pharmacodynamics of these two groups. In addition, subtle differences in the pharmacodynamics of the imidazole (ketoconazole) and the triazole (fluconazole) may account for the differences observed with the two azoles. Another important fact to mention is that fluconazole has lower efficacy against *Candida* strains compared to ketoconazole, possibly due to an increased inherent resistance of this antifungal to these isolates [39]. Resistance to azoles, both in *C. albicans* and in *C.* non-*albicans*, is caused by mechanisms that can manifest themselves individually or in combination. The main one is the high expression of transporters that expel the drug from inside the cell, the so-called efflux pumps, which lead to a very low concentration of the antifungal agent in the fungal cell, resulting in resistance to these drugs [27].

It is also important to note that 17.5% of *Candida* strains (23 isolates out of 131) evaluated in this study showed resistance to all three antifungals simultaneously. Of these, 43.5% were *C. albicans*, 21.7% *C. tropicalis*, 21.7% *C. krusei*, 8.7% *C. glabrata*, and 4.3% *C. guilliermondii* (data not shown [40]). Finding strains resistant to three antifungals increases the risks of control for patients, hospitals, and public health in general [40]. Therefore, it is essential to monitor these strains, implement rigorous infection control, and invest in the development of new antifungal drugs [41]. Furthermore, the emergence and global spread of multidrug-resistant *Candida* species, such as *C. glabrata* and the emerging pathogen *C. auris*, constitute a major concern in the care of critically ill patients [42].

In ICU settings, candidemia incidence and resistance profiles changed significantly during and after the COVID-19 pandemic, with increased frequency of *C.* non-*albicans* and higher fluconazole resistance rates, including ERG11 mutations such as Y132F in *C. parapsilosis* isolates [43,44].

It is essential to highlight that this study employed the agar disk diffusion method exclusively for antifungal susceptibility testing. Although CLSI M44 [9] supports disk diffusion as a screening approach for *Candida* spp., this method is not optimal for amphotericin B, for which interpretive criteria are limited, and clinical breakpoints are not well established. It has been reported that disk diffusion may overestimate resistance, particularly for polyenes, when compared to MIC-based methods (by the European Committee on Antibiotic Susceptibility Testing (EUCAST) and CLSI M27 methods) [45]. Therefore, the elevated resistance rates observed, especially for fluconazole and amphotericin B, should be interpreted with caution. Confirmation using quantitative MIC assays is necessary to distinguish true resistance from methodological artifacts and to enhance clinical applicability.

### 3.3. Virulence Factors

The 131 yeasts were evaluated for the presence of various virulence factors, including the production of hydrolytic enzymes (esterase, DNase, and protease), as well as hemolytic activity. The percentages of isolates of each identified species that presented the evaluated virulence factors are shown in Table 2.

Extracellular lipolytic enzymes, such as esterase, play important roles in *Candida* spp. pathogenicity, as they facilitate the pathogen’s invasion of host tissues and attack immune system cells, triggering an inflammatory process [46]. The presence of these enzymes is a determining factor in the virulence of *Candida* spp. [47]. A total of 64.9% of *Candida* strains showed esterase activity (45.8% *C. albicans*, 35.3% *C. tropicalis*, 10.6% *C. krusei*, 7.1% *C. glabrata*, and 1.2% *C. guilliermondii*). *C. parapsilosis*, *C. dubliniensis*, and *C. lusitaniae* did not present this virulence factor (Table 2). Studies have also reported higher esterase activity in *C. albicans* strains [3,48]. According to Figueiredo-Carvalho [47], it is common for *C. albicans* to have esterase activity. However, other species, such as *C. glabrata* and *C. krusei*, have also shown positive activity for esterase [49], as observed in the present study.

Regarding protease production, this is an important virulence factor for yeasts isolated from mucous membranes, as it degrades essential substrates, including immunoglobulins, skin and mucous membrane proteins, and albumin, thereby facilitating tissue invasion [50]. In this study, 45.8% of the isolates showed positive protease activity, being 35.0% of *C. albicans*, 25.0% of *C. tropicalis*, 16.7% of *C. krusei*, 13.3% of *C. glabrata*, 1.7% of *C. parapsilosis*, 5.0% of *C. dubliniensis*, 1.7% of *C. lusitaniae*, and 1.7% of *C. guilliermondii* (Table 2). There was also a greater predominance of this virulence factor in *C. albicans* isolates, as also observed in other studies [3,13,51]. Tosun and colleagues [52] report that the condition of immunosuppression in immunocompromised patients stimulates the production of protease by *C. albicans*, which can aggravate the pathogenic state caused by these yeasts.

The other virulence factor evaluated was the presence of DNase activity, which was found in 18.3% of *Candida* strains (33.3% *C. krusei*, 25.0% *C. tropicalis*, 16.7% *C. albicans*, 12.5% *C. glabrata*, 4.2% *C. parapsilosis*, and 8.3% *C. dubliniensis*) (Table 2). The species *C. lusitaniae* and *C. guilliermondii* did not present this virulence factor. Unlike the other virulence factors evaluated in this study, the prevalence of this enzyme was found in *C. krusei* strains, followed by *C. tropicalis*, but not in *C. albicans*. According to the study performed by Menezes [38], 42.0% of the *Candida* spp. presented DNase activity: *C. parapsilosis* (13 isolates, 26.0%), *C. famata* (5 isolates, 10.0%), *C. lusitaniae* (2 isolates, 4.0%), and *C. guilliermondii* (1 isolate, 2.0%). No *C. albicans* strains showed DNase activity [38]. According to these authors, DNase production in *C. albicans* is less frequent than other virulence factors.

Another important virulence factor exhibited by *Candida* spp. is hemolytic activity. As shown in Table 2, 67.2% of the *Candida* isolates showed hemolytic activity (42.0% *C. albicans*, 27.7% *C. tropicalis*, 13.6% *C. krusei*, 8.0% *C. glabrata*, 3.4% *C. parapsilosis*, 3.4% *C. dubliniensis*, and 2.3% *C. lusitaniae*). The species *C. guilliermondii* did not show this activity. A high percentage of *C. albicans* have shown hemolytic activity in different studies [13,38,48,53]. According to Rossoni [53], this can be attributed to the fact that, in addition to hemolysin, *C. albicans* produces the toxin candidalisin, which also causes the lysis of red blood cells in infections, thereby promoting hemolytic activity.

Hemolytic activity is classified according to the partial or total degradation of red blood cells. Thus, alpha (α) hemolytic activity represents partial degradation of the red blood cell, beta (β) total degradation, and gamma (γ) is classified when there is no hemolysis [3]. It was observed that 67.2% of the isolates showed hemolytic activity, with 44.3% showing partial hemolysis (α-hemolysis) and 22.9% showing total hemolysis (β-hemolysis). Among the species studied, a greater number of *C. tropicalis* strains showed β-hemolytic activity (53.3%), while *C. albicans* was more numerous in α-hemolytic activity (60.3%). Hemolytic capacity has been shown to be highly variable among *Candida* strains and species [48,54].

In general, hemolytic activity was the predominant virulence factor in this study (67.2%), followed by esterase (64.9%), protease (45.8%), and DNase (18.3%) (Table 2). According to studies, the percentage of these virulence factors in *Candida* strains varies, highlighting the importance of evaluating these characteristics in different populations and regions [3]. *C. albicans* strains displayed the majority of exoenzymatic activity, as also observed in other studies [48]. However, strains of *C. tropicalis*, *C. krusei*, and *C. glabrata* also presented all the virulence factors studied, showing the importance of evaluating *C.* non-*albicans*. Studies have shown that *C. glabrata* has become a critical cause of candidemia in recent years [55]. It is important to highlight that strains of the species *C. parapsilosis*, *C. dubliniensis*, *C. lusitaniae*, and *C. guilliermondii* also presented some evaluated virulence factors and have been considered high-priority pathogens [24,56]. A total of 120 isolates (91.6%) presented at least one of the four virulence factors investigated, and 5 strains (3.8%) presented all four virulence factors studied simultaneously.

When comparing the number of isolates of each species from the oral and tracheal cavities that present virulence factors, it is possible to verify that there is a greater number of *Candida* strains from the oral cavity presenting virulence factors than in the tracheal cavity, except for DNase (Table 3).

It has been reported that infections caused by bacteria and *Candida* spp. in the respiratory tract of patients admitted to the ICU can be caused by contamination at the time of intubation, whether through healthcare professionals, biofilms present on medical equipment, or the patient themselves, from the oral tract to the trachea [24,27]. Thus, the prevalence of strains with virulence factors in the oral cavity may represent a risk for patients undergoing invasive mechanical ventilation procedures. According to Alves [24], *Candida* spp. were isolated even after sanitization with 70% ethanol, highlighting the importance of more rigorous basic measures for controlling hospital infections to prevent nosocomial transmission. It is important to emphasize that although enzymatic activities were detected by in vitro assays, these findings should not be interpreted as a direct indicator of pathogenic potential. Virulence in *Candida* spp. is multifactorial and influenced by host–pathogen interactions, immune status, and environmental conditions that cannot be fully replicated in vitro.

In the present study, *C. albicans* strains were predominantly identified, with a significant proportion exhibiting virulence factors and resistance to antifungals. They have been reported as the primary opportunistic fungal pathogen in humans, causing a wide range of infections, and are the most frequent cause of candidemia and invasive candidiasis worldwide [55]. However, *C.* non-*albicans* have been frequently isolated and associated with pathogenic situations [22]. Thus, the yeast strains were grouped into *C. albicans* and *C.* non-*albicans* to assess whether there was an association among these groups and resistance to the antifungals (fluconazole, ketoconazole, and amphotericin B), as well as to virulence factors (esterase, DNase, protease, and hemolytic activity). A significant association (*p* < 0.05) was observed between the yeast groups and resistance to ketoconazole, as well as the presence of the virulence factors esterase and DNase (Table 4).

*C. albicans* strains were more related to resistance to ketoconazole than *C.* non-*albicans* strains (71.4% vs. 25.6%; OR = 7.26, 95% CI: 3.28–16.06; *p* < 0.001). It has been observed that the long-term, prophylactic, and indiscriminate use of this drug in the treatment of *C. albicans* infections has led to the acquisition of microbiological and clinical resistance, predominantly to ketoconazole, which has been a clinical concern [57]. The incorrect use of antifungals has favored the alteration of the epidemiological profile of fungal infections, leading to the emergence of resistant strains and increased mortality among affected patients [58,59].

Esterase activity was also more commonly detected in *C. albicans* (79.6% vs. 56.1%; OR = 3.05, 95% CI: 1.34–6.93; *p* = 0.008). In contrast, DNase activity was significantly less frequent in *C. albicans* isolates (8.2% vs. 24.4%; OR = 0.28, 95% CI: 0.09–0.86; *p* = 0.021). No statistically significant associations were observed for fluconazole resistance, amphotericin B resistance, protease activity, or hemolytic activity (Table 4). These findings indicate that, although *Candida albicans* remains the most frequently isolated species in fungal infections, non-*albicans* species can also exhibit relevant virulence attributes, including the production of DNases [47,59].

The expression of these enzymes has been associated with enhanced tissue invasion, infection persistence, and increased tolerance or resistance to antifungal agents, particularly among emerging *Candida* species [47,59]. In this context, recent studies have highlighted that microbial functional traits, such as enzymatic activity, stress adaptation, and metabolic flexibility, play a central role in determining host-microorganism interactions and microbial fitness under selective pressures, including antimicrobial exposure [54,55,56,57,58,59].

Therefore, the detection of DNase activity among non-*Candida albicans* isolates reinforces the concept that pathogenic potential is not restricted to species prevalence alone but is strongly influenced by functional and adaptive characteristics that contribute to persistence and therapeutic failure in clinical settings [58,59,60,61,62,63].

It is important to note that the observed associations between species group, ketoconazole resistance, and selected virulence factors should be interpreted cautiously. Although statistically significant, these findings are exploratory and based on effect size estimates with wide confidence intervals, reflecting sample size limitations.

The dichotomization of isolates into *C. albicans* and *C.* non-*albicans* was adopted to preserve statistical power, as several non-*albicans* species were represented by small numbers. Consequently, species-specific differences within the *C.* non-*albicans* group could not be assessed and warrant further investigation in larger, confirmatory studies

## 4. Conclusions

The results of this study demonstrated that, although chromogenic media represent a rapid and practical approach for *Candida* identification, they may fail to provide accurate species-level discrimination. Correct identification at the species level is crucial for timely diagnosis and appropriate treatment, which helps prevent drug resistance and assess the emergence of new species. *C. albicans* was the predominant species among the isolates. Hemolytic activity emerged as the most prevalent virulence factor, and high resistance rates were observed, particularly to fluconazole, followed by ketoconazole and amphotericin B. This is a significant concern, as fluconazole is widely used, and ketoconazole resistance can lead to cross-resistance with other azoles. This limits therapeutic options and may lead to treatment failures. The lower resistance to amphotericin B underscores its importance in treating severe fungal infections, despite potential adverse effects. A total of 17.5% of the *Candida* strains were resistant to all three antifungals simultaneously. This finding suggests an increased risk for patients and public health, underscoring the need for rigorous infection control and the development of new antifungal drugs.

Most isolates were recovered from the oral cavity, predominantly *C. albicans*, suggesting that this anatomical site may be an important source of yeast in ICU patients. A significant association (*p* < 0.05) was observed between yeast groups (*C. albicans* and non-*albicans*) and resistance to ketoconazole, as well as the expression of esterase and DNase. While *C. albicans* was more frequently associated with ketoconazole resistance, non-*albicans* species showed a stronger association with esterase and DNase activity. This highlights that pathogenic potential is influenced not just by species prevalence but also by functional and adaptive characteristics of isolates.

Overall, this study provides critical, clinically significant data on the epidemiology, identification, drug resistance, and virulence of *Candida* species in a specific ICU setting. It reinforces the necessity of accurate species identification, combined with assessment of virulence factors and antifungal susceptibility profiles, which are essential for understanding the pathogenic potential of *Candida* species and for supporting effective infection surveillance, antifungal stewardship, and clinical management in intensive care settings, thereby improving patient outcomes.

## 5. Limitations of the Study and Future Perspectives

The findings of this study provide a robust characterization of *Candida* species isolated from ICU patients, highlighting relevant antifungal resistance patterns and virulence traits with important clinical implications. Future investigations may expand this approach by including multicenter cohorts (multicenter studies), allowing broader comparisons across different hospital settings and epidemiological contexts.

Additional studies incorporating quantitative antifungal susceptibility methods, such as minimum inhibitory concentration (MIC) determinations, may further refine resistance profiling and provide more precise therapeutic guidance, since the present study employed only the disk diffusion method. Likewise, integrating molecular analyses to explore resistance-associated genes and the regulation of virulence factors would deepen the understanding of the pathogenic potential of both *Candida albicans* and non-*albicans* species.

An important limitation of this study is that virulence traits were assessed using qualitative assays based on the presence or absence of enzymatic activity, without semi-quantitative or quantitative scoring. Although these classical methods are widely used for initial screening of *Candida* virulence factors, the lack of standardized protocols and scoring systems across studies limits external comparability.

Given the recognized role of hyphae and biofilm formation in persistent infections and device-associated colonization in intensive care units, future research should also address the relationship between hyphae and biofilm production, antifungal resistance, and virulence expression. Moreover, linking microbiological findings with clinical data, such as antifungal exposure, duration of mechanical ventilation, and patient outcomes, may strengthen translational insights and support antifungal stewardship strategies, which was a limitation of this study.

Overall, the present study establishes a valuable foundation for future investigations aimed at improving surveillance, infection control practices, and therapeutic decision-making in critical care settings.

## Figures and Tables

**Table 1:** Antifungal resistance of Candida species isolated from the oral and tracheal cavities of patients admitted to the ICU.

Species	Antifungal Resistance *					
	Fluconazole		Ketoconazole		Amphotericin B	
	N	%	N	%	N	%
Oral Cavity						
*C. albicans*	21	50.0	26	74.3	08	53.3
*C. tropicalis*	07	16.7	03	8.6	03	20.0
*C. krusei*	11	26.2	04	11.4	02	13.3
*C. glabrata*	03	7.1	02	5.7	01	6.7
*C. parapsilosis*	-	-	-	-	01	6.7
*C. dubliniensis*	00	00	00	00	-	-
*C. lusitaniae*	-	-	-	-	-	-
*C. guilliermondii*	00	00	00	00	00	00
Total	42	32.1 **	35	26.6 **	15	11.5 **
Tracheal Cavity						
*C. albicans*	07	28.0	09	42.9	02	20.0
*C. tropicalis*	06	24.0	04	19.0	03	30.0
*C. krusei*	06	24.0	04	19.0	03	30.0
*C. glabrata*	04	16.0	02	9.5	01	10.0
*C. parapsilosis*	-	-	-	-	00	00
*C. dubliniensis*	01	4.0	01	4.8	-	-
*C. lusitaniae*	-	-	-	-	-	-
*C. guilliermondii*	01	4.0	01	4.8	01	10.0
Total	25	19.1 **	21	16 **	10	7.7 **

* Percentage of isolates that showed resistance in relation to the total number of isolates resistant to the respective antifungal (Fluconazole: n = 67; Ketoconazole: n = 56; Amphotericin B: n = 25). ** Percentage of isolates that showed antifungal resistance in relation to the total number of isolates (n = 131).

**Table 2:** Virulence factors of *Candida* strains isolated from patients admitted to the ICU.

Species	Virulence Factors *							
	Esterase		DNase		Protease		Hemolytic Activity	
	N	%	N	%	N	%	N	%
*C. albicans*	39	45.8	04	16.7	21	35.0	37	42.0
*C.tropicalis*	30	35.3	06	25.0	15	25.0	24	27.7
*C. krusei*	09	10.6	08	33.3	10	16.7	12	13.6
*C. glabrata*	06	7.1	03	12.5	08	13.3	07	8.0
*C. parapsilosis*	-	-	01	4.2	01	1.7	03	3.4
*C. dubliniensis*	-	-	02	8.3	03	5.0	03	3.4
*C. lusitaniae*	-	-	-	-	01	1.7	02	2.3
*C. guilliermondii*	01	1.2	-	-	01	1.7	-	-
Total	85	64.9 **	24	18.3 **	60	45.8 **	88	67.2 **

* Percentage of isolates that showed virulence in relation to the total number of virulent yeasts in the respective test (Esterase: n = 85; DNase: n = 24; Protease: n = 60; Hemolytic Activity: n = 88). ** Percentage of isolates that showed virulence in relation to the total number of yeasts (n = 131).

**Table 3:** Virulence factors of *Candida* strains present in the oral and tracheal cavities isolated from patients admitted to the ICU.

Species	Virulence Factors *							
	Esterase		DNase		Protease		Hemolytic Activity	
	N	%	N	%	N	%	N	%
Oral cavity								
*C. albicans*	27	51.9	03	33.3	14	34.1	26	48.1
*C.tropicalis*	17	32.7	00	00	12	29.3	14	25.9
*C. krusei*	05	9.6	05	55.6	08	19.5	07	13.0
*C. glabrata*	03	5.8	00	00	05	12.2	03	5.5
*C. parapsilosis*	-	-	00	00	00	00	01	1.9
*C. dubliniensis*	-	-	01	11.1	02	4.9	02	3.7
*C. lusitaniae*	-	-	-	-	00	00	01	1.9
*C. guilliermondii*	00	00	-	-	00	00	-	-
Total	52	39.7 **	09	6.9 **	41	31.3 **	54	41.2 **
Tracheal cavity								
*C. albicans*	12	36.4	01	6.6	07	36.8	11	32.4
*C.tropicalis*	13	39.4	06	40.0	03	15.8	10	29.4
*C. krusei*	04	12.1	03	20.0	02	10.4	05	14.7
*C. glabrata*	03	9.1	03	20.0	03	15.8	04	11.8
*C. parapsilosis*	-	-	01	6.7	01	5.3	02	5.9
*C. dubliniensis*	-	-	01	6.7	01	5.3	01	2.9
*C. lusitaniae*	-	-	-	-	01	5.3	01	2.9
*C. guilliermondii*	01	3.0	-	-	01	5.3	-	-
Total	33	25.2 **	15	11.4 **	19	14.5 **	34	26.0 **

* Percentage of isolates that showed virulence in relation to the total number of virulent yeasts in the respective test (Esterase: n = 85; DNase: n = 24; Protease: n = 60; Hemolytic Activity: n = 88). ** Percentage of isolates that showed virulence in relation to the total number of yeasts (n = 131).

**Table 4:** Association of *C. albicans* and *C.* non-*albicans* with resistance to commercial antifungals and virulence factors.

	*C. albicans*	*C.* non-*albicans*	OR (95% CI)	*p* Value *
Fluconazole resistance	28/49 (57.1)	39/82 (47.6)	1.47 (0.72–3.00)	0.29
Ketoconazole resistance	35/49 (71.4)	21/82 (25.6)	7.26 (3.28–16.06)	0.00
Amphotericin B resistance	10/49 (20.4)	15/82 (18.3)	1.14 (0.47–2.77)	0.77
Esterase activity	39/49 (79.6)	46/82 (56.1)	3.05 (1.34–6.93)	0.01
DNase activity	4/49 (8.2)	20/82 (24.4)	0.28 (0.09–0.86)	0.02 **
Protease activity	21/49 (42.9)	39/82 (47.6)	0.83 (0.41–1.68)	0.60
Hemolytic activity	37/49 (75.5)	51/82 (62.2)	1.87 (0.85–4.13)	0.12

* *p* < 0.05: statistically significant association according to the chi-square test.** *p* < 0.05: statistically significant association according to the Fisher test.

## Data Availability

The original contributions presented in this study are included in the article. Further inquiries can be directed to the corresponding authors.

## References

[1] Pfaller M.A., Diekema D.J., Turnidge J.D., Castanheira M., Jones R.N. (2019). Twenty years of the SENTRY antifungal surveillance program: Results for *Candida* species from 1997–2016. Open Forum Infect. Dis..

[2] Staniszewska M. (2020). Virulence factors in *Candida* species. Curr. Protein Pept. Sci..

[3] Musinguzi B., Akampurira A., Derick H., Mwesigwa A., Mwebesa E., Mwesigye V., Kabajulizi I., Sekulima T., Ocheng F., Itabangi H. (2025). Extracellular hydrolytic enzyme activities and biofilm formation in *Candida* species isolated from people living with human immunodeficiency virus with oropharyngeal candidiasis at HIV/AIDS clinics in Uganda. Microb. Pathog..

[4] Lee C., Sun L., Gounder A.P., Watanabe M., Marrero L., Kobayashi H., Ainslie K.M., Brown G.D., Wong M.H., Underhill D.M. (2021). Structural specificities of cell surface β-glucan polysaccharides determine commensal yeast–mediated immunomodulatory activities. Nat. Commun..

[5] Akimowicz M., Bucka-Kolendo J. (2020). MALDI-TOF MS—Application in food microbiology. Acta Biochim. Pol..

[6] Lockhart S.R., Lyman M.M., Sexton D.J. (2022). Tools for Detecting a “Superbug”: Updates on *Candida auris* Testing. J. Clin. Microbiol..

[7] Ferreira P.H.d.C., Moura C.R.F., Lage V.K.S., Teixeira L.A.C., Costa H.S., Figueiredo P.H.S., Santos J.N.V., de Jesus P.H.C., Freitas D.A., Lacerda A.C.R. (2025). Factors associated with the presence of *Candida* spp. in oral and tracheobronchial secretions of adult intensive care unit patients. Diagn. Microbiol. Infect. Dis..

[8] Nielsen D.S., Teniola O.D., Ban-Koffi L., Owusu M., Andersson T.S., Holzapfel W.H. (2007). The microbiology of Ghanaian cocoa fermentations analysed using culture-dependent and culture-independent methods. Int. J. Food Microbiol..

[9] Clinical and Laboratory Standards Institute (2018). Method for Antifungal Disk Diffusion Susceptibility Testing of Yeasts; CLSI Guideline M44.

[10] Galán-Ladero M.A., Blanco M.T., Sacristán B., Fernández-Calderón M.C., Pérez-Giraldo C., Gómez-García A.C. (2010). Enzymatic activities of *Candida tropicalis* isolated from hospitalized patients. Med. Mycol..

[11] Riceto É.B.d.M., Menezes R.P., Penatti M.P.A., Pedroso R.d.S. (2015). Enzymatic and hemolytic activity in different *Candida* species. Rev. Iberoam. Micol..

[12] Staib F. (1966). Serum proteins as a nitrogen source for yeast-like fungi. Sabouraudia.

[13] Rörig K.C.O., Colacite J., Abegg M.A. (2009). Production of virulence factors in vitro by pathogenic species of the genus *Candida*. Rev. Soc. Bras. Med. Trop..

[14] Hulimane S., Maluvadi-Krishnappa R., Mulki S., Rai H., Dayakar A., Kabbinahalli M. (2018). Speciation of *Candida* using CHROMagar in cases with oral epithelial dysplasia and squamous cell carcinoma. J. Clin. Exp. Dent..

[15] Odds F.C., Bernaerts R. (1994). CHROMagar *Candida*, a new differential isolation medium for presumptive identification of clinically important *Candida* species. J. Clin. Microbiol..

[16] Pfaller M.A., Houston A., Coffmann S. (1996). Application of CHROMagar *Candida* for rapid screening of clinical isolates. J. Clin. Microbiol..

[17] García-Salazar E., Betancourt-Cisneros P., Ramírez-Magaña X., Díaz-Huerta H., Martínez-Herrera E., Frías-De-León M.G. (2025). Utility of Cand PCR in the diagnosis of vulvovaginal candidiasis in pregnant women. J. Fungi.

[18] Menu E., Criscuolo A., Desnos-Ollivier M., Cassagne C., D’Incan E., Furst S., Ranque S., Berger P., Dromer F. (2020). Outbreak of Saprochaete clavata Infecting a Cancer Center Through a Dish-Washing Machine. Emerg. Infect. Dis..

[19] Parambath S., Dao A., Kim H.Y., Zawahir S., Izquierdo A.A., Tacconelli E., Govender N., Oladele R., Colombo A., Sorrell T. (2024). *Candida albicans*—A systematic review to inform the World Health Organization Fungal Priority Pathogens List. Med. Mycol..

[20] Barbedo L.S., Sgarbi D.B.G. (2010). Candidiasis. J. Bras. Doenças Sex. Transm..

[21] Lohse M.B., Gulati M., Johnson A.D., Nobile C.J. (2018). Development and regulation of single- and multi-species *Candida albicans* biofilms. Nat. Rev. Microbiol..

[22] Tortorano A.M., Prigitano A., Morroni G., Brescini L., Barchiesi F. (2021). Candidemia: Evolution of drug resistance and novel therapeutic approaches. Infect. Drug Resist..

[23] Pfaller M.A., Diekema D.J., Gibbs D.L., Newell V.A., Ellis D., Tullio V., Rodloff A., Fu W., Ling T.A. (2010). The Global Antifungal Surveillance Group. Results from the ARTEMIS DISK Global Antifungal Surveillance Study, 1997–2007: A 10.5-year analysis of susceptibilities of *Candida* species to fluconazole and voriconazole as determined by CLSI standardized disk diffusion. J. Clin. Microbiol..

[24] Alves P.G.V., Menezes R.P., Silva N.B.S., Faria G.O., Bessa M.A.S., Araújo L.B., Aguiar P.A.D.F., Penatti M.P.A., Pedroso R.d.S., Röder D.v.D.B. (2024). Virulence factors, antifungal susceptibility and molecular profile in *Candida* species isolated from the hands of health professionals before and after cleaning with 70% ethyl alcohol–based gel. J. Mycol. Med..

[25] Lopes J.P., Lionakis M.S. (2022). Pathogenesis and virulence of *Candida albicans*. Virulence.

[26] Wang X., Wu S., Li L., Yan Z. (2023). *Candida albicans* overgrowth disrupts the gut microbiota in mice bearing oral cancer. Mycology.

[27] Govrins M., Lass-Flörl C. (2024). *Candida parapsilosis* complex in the clinical setting. Nat. Rev. Microbiol..

[28] McHugh J.W., Bayless D.R., Ranganath N., Stevens R.W., Kind D.R., Wengenack N.L., Shah A.S. (2024). *Candida guilliermondii* fungemia: A 12-year retrospective review of antimicrobial susceptibility patterns at a reference laboratory and tertiary care center. J. Clin. Microbiol..

[29] Lima Y.P., Bastos L.Q.D.A., Bastos V.Q.D.A., Bastos R.V., Bastos Y.S., Bastos A.N., Machado A.B.F., Watanabe A.A.S., Diniz C.G., Silva V.L. (2024). Epidemiologia das infecções por *Candida* spp. em pacientes comunitários e hospitalizados em Juiz de Fora, MG. Doenças Infecciosas e Parasitárias.

[30] Moloi M., Lenetha G.G., Malebo N.J. (2021). Microbial levels on street foods and food preparation surfaces in Mangaung Metropolitan Municipality. Health SA Gesondheid.

[31] Silva S.L., Lima M.E., dos Santos R.D.T., Lima E.O. (2020). Onychomycoses due to fungi of the genus *Candida*: A literature review. Res. Soc. Dev..

[32] Gamaletsou M.N., Rammaert B., Bueno M.A., Sipsas N.V., Moriyama B., Kontoyiannis D.P., Roilides E., Zeller V., Taj Aldeen S.J., Miller A.O. (2015). *Candida* arthritis: Analysis of 112 pediatric and adult cases. Open Forum Infect. Dis..

[33] Zheng L., Xu Y., Wang C., Guo L. (2024). Ketoconazole induces reversible antifungal drug tolerance mediated by trisomy of chromosome R in *Candida albicans*. Front. Microbiol..

[34] Masala L., Luzzati R., Maccacaro L., Antozzi L., Concia E., Fontana R. (2003). Nosocomial cluster of *Candida guilliermondii* fungemia in surgical patients. Eur. J. Clin. Microbiol. Infect. Dis..

[35] Dick J.D., Rosengard B.R., Merz W.G., Stuart R.K., Hutchins G.M., Saral R. (1985). Fatal disseminated candidiasis due to amphotericin B–resistant *Candida guilliermondii*. Ann. Intern. Med..

[36] Seneviratne C.J., Rajan S., Wong S.S., Tsang D.N., Lai C.K., Samaranayake L.P., Jin L. (2016). Antifungal susceptibility in serum and virulence determinants of *Candida* bloodstream isolates from Hong Kong. Front. Microbiol..

[37] Savastano C., Silva E.O., Gonçalves L.L., Nery J.M., Silva N.C., Dias A.L. (2016). *Candida glabrata* among *Candida* spp. from environmental health practitioners of a Brazilian hospital. Braz. J. Microbiol..

[38] de Paula Menezes R., de Melo Riceto É.B., Borges A.S., de Brito Röder D.V.D., dos Santos Pedroso R. (2016). Evaluation of virulence factors of *Candida albicans* isolated from HIV-positive individuals using HAART. Arch. Oral Biol..

[39] Ellepola A.N.B., Chandy R., Khan Z.U. (2015). Post-antifungal effect and adhesion to buccal epithelial cells of oral *Candida dubliniensis* isolates subsequent to limited exposure to amphotericin B, ketoconazole and fluconazole. J. Investig. Clin. Dent..

[40] Jensen R.H., Astvad K.M.T., Silva L.V., Sanglard D., Jørgensen R., Nielsen K.F., Mathiasen E.G., Doroudian G., Perlin D.S., Arendrup M.C. (2015). Stepwise emergence of azole, echinocandin and amphotericin B multidrug resistance in vivo in *Candida albicans* orchestrated by multiple genetic alterations. J. Antimicrob. Chemother..

[41] Fisher M.C., Hawkins N.J., Sanglard D., Gurr S.J. (2022). Tackling the emerging threat of antifungal resistance in *Candida* spp.. Nat. Rev. Microbiol..

[42] Cortegiani A., Misseri G., Chowdhary A. (2019). What’s new on emerging resistant *Candida* species. Intensive Care Med..

[43] Nucci M., Barreiros G., Guimarães L.F., Deriquehem V.A.S., Castiñeiras A.C., Nouér S.A. (2021). Increased incidence of candidemia in a tertiary care hospital with the COVID-19 pandemic. Mycoses.

[44] Arastehfar A., Daneshnia F., Hilmioğlu-Polat S., Fang W., Yaşar M., Polat F., Metin D.Y., Rigole P., Coenye T., Ilkit M. (2020). First Report of Candidemia Clonal Outbreak Caused by Emerging Fluconazole-Resistant *Candida parapsilosis* Isolates Harboring Y132F and/or Y132F+K143R in Turkey. Antimicrob Agents Chemother..

[45] Arendrup M.C., Guinea J., Arikan-Akdagli S., Meijer E.F.J., Meis J.F., Buil J.B., Dannaoui E., Giske C.G., Lyskova P., Meletiadis J. (2026). How to interpret MICs of amphotericin B, echinocandins and flucytosine against *Candida auris* (*Candidozyma auris*) according to the newly established European Committee for Antimicrobial Susceptibility Testing (EUCAST) breakpoints. Clin. Microbiol. Infect..

[46] Ramos L.S., Branquinha M.H., Santos A.L.S. (2017). Different classes of hydrolytic enzymes produced by multidrug-resistant yeasts comprising the *Candida haemulonii* complex. Med. Mycol..

[47] Figueiredo-Carvalho M.H.G., Silva R.T., Souza A.A., Oliveira J.A., Silva T.A., Pereira M.D., Santos L.M., Almeida M.F., Lima A.P., Costa A.L. (2017). Relationship between the antifungal susceptibility profile and the production of virulence-related hydrolytic enzymes in Brazilian clinical strains of *Candida glabrata*. Mediat. Inflamm..

[48] Nouraei H., Ghaderian Jahromi M., Razeghian Jahromi L., Zomorodian K., Pakshir K. (2021). Potential pathogenicity of *Candida* species isolated from oral cavity of patients with diabetes mellitus. Biomed. Res. Int..

[49] Kalaiarasan K., Singh R., Chaturvedula L. (2018). Changing virulence factors among vaginal non-albicans *Candida* species. Indian J. Med. Microbiol..

[50] Junqueira J.C., Vilela S.F.G., Rossoni R.D., Barbosa J.O., Costa A.C.B.P., Rasteiro V.M.C., Suleiman J.M.A.H., Jorge A.O.C. (2012). Oral colonization by yeasts in HIV-positive patients in Brazil. Rev. Inst. Med. Trop. Sao Paulo.

[51] Makled A.F., Ali S.A.M., Labeeb A.Z., El-Sayed M.A., Hassan Z.K., Abdel-Hakim A., Ahmed A.M., Sabal M.S. (2024). Characterization of *Candida* species isolated from clinical specimens: Insights into virulence traits, antifungal resistance and molecular profiles. BMC Microbiol..

[52] Tosun İ., Aydın F., Kaklıkaya N., Ertürk M. (2005). Induction of secretory aspartyl proteinase of *Candida albicans* by HIV-1 but not HSV-2 or some other microorganisms associated with vaginal environment. Mycopathologia.

[53] Rossoni R.D., Barbosa J.O., Vilela S.F.G., Jorge A.O.C., Junqueira J.C. (2013). Comparison of the hemolytic activity between *Candida albicans* and non-albicans *Candida* species. Rev. Iberoam. Micol..

[54] Furlaneto M.C., Góes H.P., Perini H.F., dos Santos R.C., Furlaneto-Maia L. (2018). How much do we know about hemolytic capability of pathogenic *Candida* species?. Folia Microbiol..

[55] Katsipoulaki M., Stappers M.H.T., Malavia-Jones D., Brunke S., Hube B., Gow N.A.R. (2024). *Candida albicans* and *Candida glabrata*: Global priority pathogens. Microbiol. Mol. Biol. Rev..

[56] Rajni E., Chaudhary P., Garg V.K., Sharma R., Malik M. (2022). A complete clinico-epidemiological and microbiological profile of candidemia cases in a tertiary-care hospital in Western India. Antimicrob. Steward Healthc. Epidemiol..

[57] Siikala E., Richardson M., Pfaller M.A., Diekema D.J., Messer S.A., Perheentupa J., Saxén H., Rautemaa R. (2009). *Candida albicans* isolates from APECED patients show decreased susceptibility to miconazole. Int. J. Antimicrob. Agents.

[58] Fernandes G.Q., Magalhães J.C.S. (2021). Profile of resistance of opportunistic mycosis agents in Brazil. InterAm. J. Med. Health.

[59] Schnabl J.T., Sandini S., Stärkel P., Hartmann P. (2025). Serum Levels of *Candida albicans* 65-kDa Mannoprotein (CaMp65p) Correlate with Liver Disease in Patients with Alcohol Use Disorder. Microorganisms.

[60] Magalhães K.T. (2025). Probiotic-Fermented Foods and Antimicrobial Stewardship: Mechanisms, Evidence, and Translational Pathways Against AMR. Fermentation.

[61] Sasani E., Yadegari M.H., Khodavaisy S., Rezaie S., Salehi M., Getso M.I. (2021). Virulence factors and azole-resistant mechanism of *Candida tropicalis* isolated from candidemia. Mycopathologia.

[62] Magalhães K.T., da Silva R.N.A., Borges A.S., Siqueira A.E.B., Puerari C., Bento J.A.C. (2025). Smart and Functional Probiotic Microorganisms: Emerging Roles in Health-Oriented Fermentation. Fermentation.

[63] Gao Y., Liu S., Wang J., Xu Y., Guo Y., Fang Z., Wang F., Luo J., Yan L. (2025). Effects of Cinnamon Essential Oil on Intestinal Flora Regulation of Ulcerative Colitis Mice Colonized by *Candida albicans*. Microorganisms.

